# Flower-Specific Overproduction of Cytokinins Altered Flower Development and Sex Expression in the Perennial Woody Plant *Jatropha curcas* L.

**DOI:** 10.3390/ijms21020640

**Published:** 2020-01-18

**Authors:** Xin Ming, Yan-Bin Tao, Qiantang Fu, Mingyong Tang, Huiying He, Mao-Sheng Chen, Bang-Zhen Pan, Zeng-Fu Xu

**Affiliations:** 1 School of Life Sciences, University of Science and Technology of China, Hefei 230027, China; mx007@mail.ustc.edu.cn; 2 CAS Key Laboratory of Tropical Plant Resources and Sustainable Use, Xishuangbanna Tropical Botanical Garden, The Innovative Academy of Seed Design, Chinese Academy of Sciences, Menglun, Mengla 666303, Chinatangmingyong@xtbg.ac.cn (M.T.); hhy@xtbg.org.cn (H.H.); chenms@xtbg.org.cn (M.-S.C.); pbz@xtbg.org.cn (B.-Z.P.); 3 Center of Economic Botany, Core Botanical Gardens, Chinese Academy of Sciences, Menglun, Mengla 666303, China

**Keywords:** cytokinin, *AtIPT4*, *JcTM6* promoter, flower development, bisexual flower, *Jatropha curcas* L.

## Abstract

*Jatropha curcas* L. is monoecious with a low female-to-male ratio, which is one of the factors restricting its seed yield. Because the phytohormone cytokinins play an essential role in flower development, particularly pistil development, in this study, we elevated the cytokinin levels in *J. curcas* flowers through transgenic expression of a cytokinin biosynthetic gene (*AtIPT4*) from Arabidopsis under the control of a *J. curcas* orthologue of *TOMATO MADS BOX GENE 6* (*JcTM6*) promoter that is predominantly active in flowers. As expected, the levels of six cytokinin species in the inflorescences were elevated, and flower development was modified without any alterations in vegetative growth. In the transgenic *J. curcas* plants, the flower number per inflorescence was significantly increased, and most flowers were pistil-predominantly bisexual, i.e., the flowers had a huge pistil surrounded with small stamens. Unfortunately, both the male and the bisexual flowers of transgenic *J. curcas* were infertile, which might have resulted from the continuously high expression of the transgene during flower development. However, the number and position of floral organs in the transgenic flowers were well defined, which suggested that the determinacy of the floral meristem was not affected. These results suggest that fine-tuning the endogenous cytokinins can increase the flower number and the female-to-male ratio in *J. curcas*.

## 1. Introduction

The monoecious *Jatropha curcas* L., which belongs to the *Euphorbiaceae* family, is a perennial woody plant that is considered a promising feedstock for biodiesel production because it contains a high amount of seed oil [1,2,3]. However, the low seed yield of this plant, which is most likely caused by the small number of female flowers, restricts its application in the biodiesel industry [4,5]. Therefore, the manipulation of flower development to increase the number of female flowers is critical for *J. curcas* breeding.

The phytohormone cytokinins play an important role in the regulation of flower development, and their content and distribution are critical for guaranteeing normal flower development [6,7,8]. The initial and rate-limiting step in cytokinin biosynthesis is catalyzed by isopentenyltransferases (IPTs) [9,10], and LONELY GUY (LOG) directly converts the nucleotide precursors of cytokinins to the biologically active forms at the last step [11]. The degeneration of cytokinin is catalyzed by cytokinin oxidases/dehydrogenases (CKXs) [12]. In Arabidopsis, nine *IPT* genes (*AtIPT1* to *AtIPT9*) have been identified, and their vital functions in cytokinin biosynthesis have been studied [13,14,15,16,17]. The expression of *AtIPT4* in Arabidopsis under the control of the flower-specific *APETALA1* (*AP1*) promoter increased the flower numbers and altered the morphology of the flowers [18], and the *ckx3 ckx5* mutant exhibited a similar phenotype [19]. In rice, the downregulation of *OsCKX2* increased the accumulation of cytokinins and the reproductive meristem, resulting in an enhanced grain yield [20,21]. Accordingly, cytokinin-deficient log3 log4 log7 triple mutant and *AtCKX-*overexpressing transgenic Arabidopsis produce only a small number of flowers [12,22]. Thus, the flower number can be controlled by modulating the cytokinin levels.

At the early flower developmental stage of a meristem-organizing center to be formed, cytokinins establish the meristematic competence of the floral meristem [23]. During pistil establishment, the cytokinin signaling B-type regulators ARABIDOPSIS RESPONSE REGULATOR (ARR)1 and ARR10 bind to the promoter of *AGAMOUS* (*AG*) and thereby induce expression of this carpel identity gene [24]. In addition, the pistils were absent in rice *log* mutant, in which active cytokinin levels were reduced [11], and absence of the *SHOOT MERISTEMLESS* (*STM*) gene, an activator of cytokinin biosynthesis, in Arabidopsis also results in the production of flowers without pistils. Ectopic pistils are produced in plants overexpressing the *STM* [25,26,27]. The female fertility of cytokinin receptor triple mutant *ahk2 3 4* Arabidopsis is impaired [28,29], and the gynoecium in the *arr1 10 12* triple mutant shows defects in septum fusion [30]. In *J. curcas*, the concentration of zeatin in female flowers is higher than that in male flowers at the early stage of flower development [31]. *J. curcas JcARR6*, an orthologue of the cytokinin response gene *ARR6* of Arabidopsis, is significantly upregulated in female compared with male flowers at the initial stage of flower development [32]. Moreover, the expression levels of the cytokinin biosynthetic genes *JcIPT5*, *JcCYP735A1,* and *JcLOG5* were higher in gynoecious *J. curcas* with degenerated male flowers than in monoecious *J. curcas* [33].

Because cytokinin is critical for pistil development [6,23], it is considered a major phytohormone responsible for expression of the female sex [34]. Therefore, the exogenous application of cytokinin is usually used to promote floral feminization in many plant species, such as *Vitis vinifera* [35], *Spinacia oleracea* [36], *Mercurialis annua* [37], *Plukenetia volubilis* [38], and *Sapium sebiferum* [39]. Notably, in *J. curcas*, the application of synthetic cytokinins to inflorescences significantly elevates the female-to-male ratio, resulting in an increase in seed yield [40,41]. We thus hypothesize that cytokinin has the potential to be a key driver of modifying flower traits in *J. curcas* through genetic manipulation. As in canola, the transgenic expression of *ipt* under the control of the *AtMYB32* promoter produces more flowers and increases the seed yield [42].

In this study, to investigate the effect of endogenous cytokinins on flower development in *J. curcas*, we elevated the levels of cytokinins specifically in flowers through the transgenic expression of *AtIPT4* under the control of a *J. curcas* orthologue *TOMATO MADS BOX GENE 6* (*JcTM6*) promoter, which is predominantly active in *J. curcas* flowers. The increase in endogenous cytokinins in transgenic *J. curcas* resulted in the production of a notably higher number of flowers with an increased proportion of bisexual flowers. This finding suggests that cytokinins can enhance the inflorescence meristem, resulting in the formation of more flowers and alterations in sex expression in *J. curcas*.

## 2. Results

### 2.1. Characterization of the JcTM6 Promoter in J. curcas

To specifically express the transgene in *J. curcas* flowers, we isolated a 1.8-kb promoter fragment (GenBank accession no. MN044579) from *JcTM6*, which is an orthologue of tomato *TM6* that is specifically expressed in flowers [43]. The *JcTM6* promoter activity was examined through a β-glucuronidase (GUS) assay of transgenic *J. curcas* harboring the *JcTM6:GUS* fusion.

**Figure 1:**
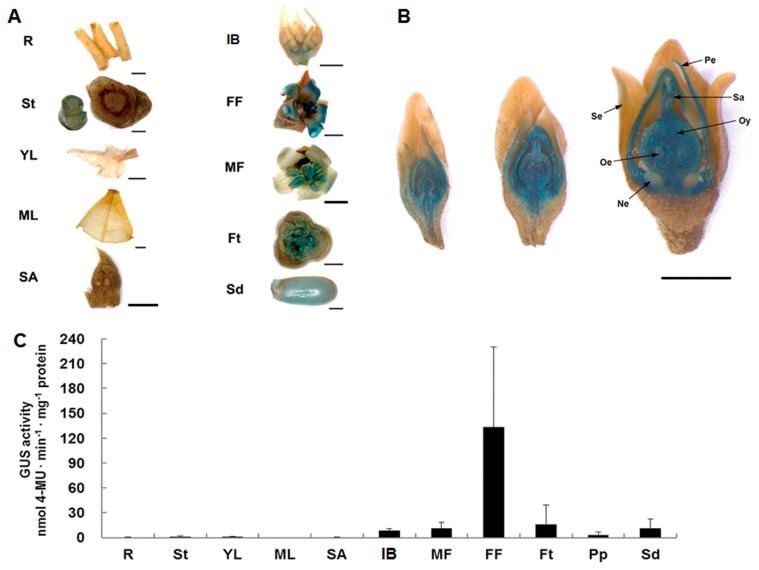
Analysis of the activity of the *J. curcas* orthologue of *TOMATO MADS BOX GENE 6* (*JcTM6*) promoter in transgenic *J. curcas*. (**A**) Histochemical β-glucuronidase (GUS) staining in various plant organs of adult transgenic *J. curcas* plants (T0). (**B**) Histochemical GUS staining in female flowers during development. (**C**) Fluorometric assay of GUS activity in adult transgenic *J. curcas* plants (T0). The values represent the means ± standard deviations (*n* = 5). The GUS activities were measured three times. FF, female flowers; Ft, fruits at 12 days after pollination (DAP); IB, inflorescence buds; MF, male flowers; ML, mature leaves; Ne, nectaries; Oe, ovules; Oy, ovaries; Pe, petals; Pp, pericarps at 25 DAP; R, roots; SA, shoot apices; Sa, stigmas; Sd, seeds at 25 DAP; Se, sepals; St, stems; and YL, young leaves. Scale bars = 2 mm.

Various plant organs of transgenic *J. curcas,* including the roots, stems, young and mature leaves, shoot apices, inflorescence buds, female and male flowers, fruits at 12 days after pollination (DAP), and seeds at 25 DAP, were evaluated through a histochemical GUS assay. As shown in Figure 1A, GUS activity was mostly detected in female flowers, which presented strong staining, whereas no activity was observed in vegetative plant organs. Strong GUS staining was observed during female flower development (Figure 1B). Furthermore, we compared the activity of the *JcTM6* promoter in these plant organs through a fluorometric GUS assay. Plant organs from five independent transgenic lines were used in this analysis. The results (Figure 1C) showed that GUS expression was predominantly detected in female flowers, followed by inflorescence buds, male flowers, fruits at 12 DAP and seeds at 25 DAP, which is consistent with the GUS staining results. These results indicate that the *JcTM6* promoter is active in reproductive plant organs, particularly in female flowers.

### 2.2. Cytokinin Contents in JcTM6:AtIPT4 Transgenic J. curcas

**Figure 2:**
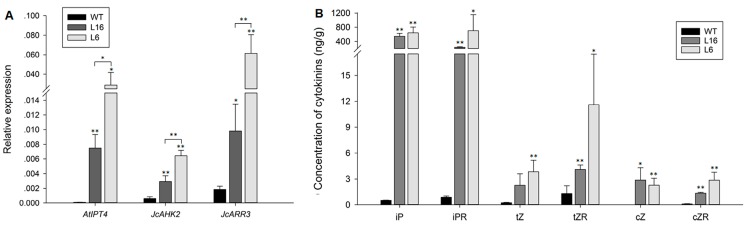
The cytokinin contents were increased in *JcTM6:AtIPT4* transgenic *J. curcas*. (**A**) Relative expression of *AtIPT4*, *JcAHK2,* and *JcARR3* in the inflorescence buds of the wild-type (WT) and transgenic *J. curcas* plants. The levels were normalized using the amplified products of *JcAct1*. The values are presented as the means ± standard deviations (*n* = 3). (**B**) Six cytokinin species in the inflorescence buds of transgenic and WT plants were examined. iP, isopentenyladenine; iPR, isopentenyladenosine; tZ, trans-zeatin; tZR, trans-zeatin riboside; cZ, cis-zeatin; and cZR, cis-zeatin riboside. The values represent the means ± standard deviations (*n* = 3). Student’s *t*-test was used for the statistical analyses: * *p* ≤ 0.05, ** *p* ≤ 0.01.

To study the effect of cytokinins on *J. curcas* flowers and reproductive floral organ development, we transformed *J. curcas* with the Arabidopsis cytokinin biosynthetic gene *AtIPT4* under the control of the *JcTM6* promoter. We successfully generated 15 independent transgenic *J. curcas* lines, which were identified through the detection of *AtIPT4* expression in the inflorescence buds, where the *JcTM6* promoter was active (Figure 1A). The endogenous levels of six cytokinin species, isopentenyladenine (iP), isopentenyladenosine (iPR), trans-zeatin (tZ), trans-zeatin riboside (tZR), cis-zeatin (cZ), and cis-zeatin riboside (cZR), in the inflorescence buds of wild-type (WT) plants and two independent transgenic lines, L16 and L6, which respectively exhibited intermediate and high expression levels of *AtIPT4* and cytokinin signaling genes *JcAHK2* and *JcARR3* (Figure 2A), were examined. As shown in Figure 2B, the levels of all six cytokinin species were significantly increased in the transgenic lines. Among the examined cytokinin species, the levels of iP-type cytokinins showed the highest increases, which suggests that IPT is more effective for the production of iP-type cytokinins in *J. curcas*.

### 2.3. The Flower Number was Increased in JcTM6:AtIPT4 Transgenic J. curcas

**Figure 3:**
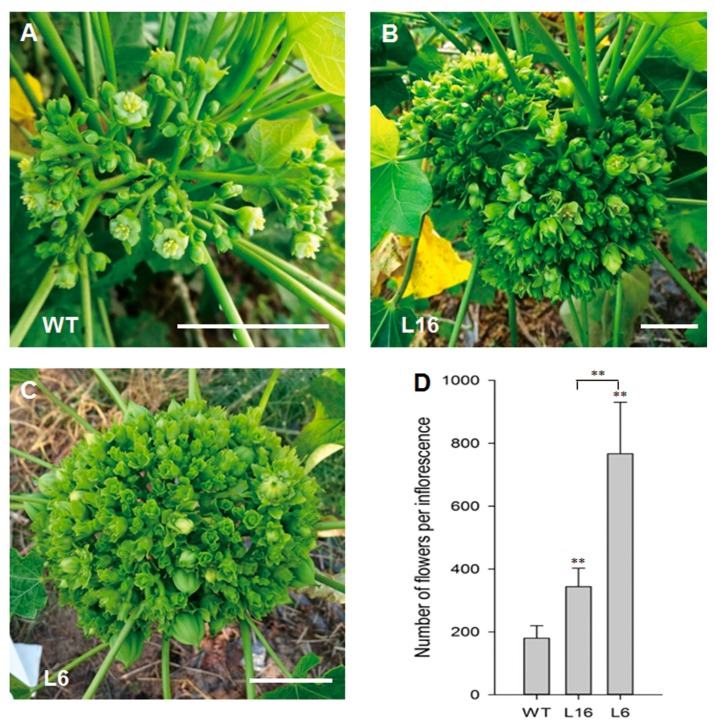
The flower number per inflorescence was increased in *JcTM6:AtIPT4* transgenic *J. curcas*. (**A**–**C**) Inflorescences of WT plants (**A**) and the transgenic lines L16 (**B**) and L6 (**C**); scale bars = 3 cm. (**D**) Number of flowers per inflorescence in WT and transgenic *J. curcas*. The values represent the means ± standard deviations (*n* = 8). Student’s *t*-test was used for the statistical analyses: ** *p* ≤ 0.01.

As expected, the transgenic *J. curcas* plants showed morphological changes in their flowers without any alterations in vegetative growth. Compared with the WT plants, all the transgenic plants produced a notably higher number of flowers (Figure 3A–C). The average numbers of flowers per inflorescence in the transgenic lines L16 and L6 were 344 and 767, respectively, whereas the corresponding number in the WT plants was 180 (Figure 3D). These results indicate that the elevated cytokinin contents in transgenic *J. curcas* induce inflorescence buds to form a higher number of flowers.

### 2.4. JcTM6:AtIPT4 transgenic J. curcas Produced Bisexual Flowers

**Figure 4:**
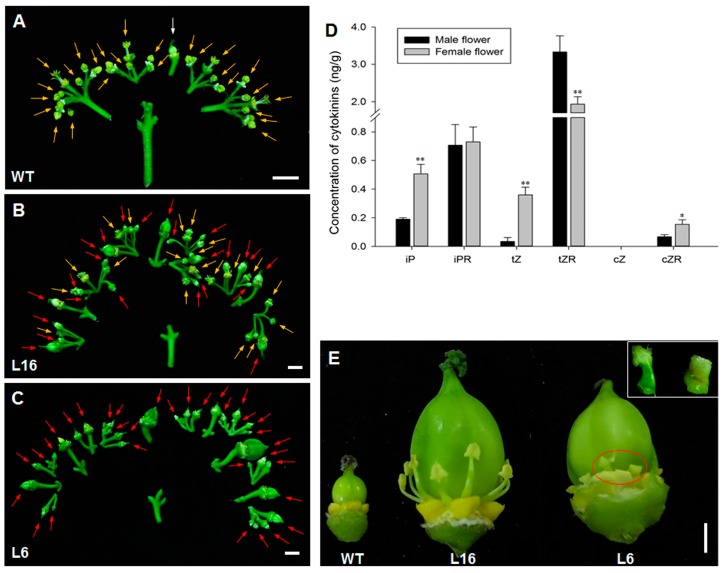
*JcTM6:AtIPT4* transgenic *J. curcas* produced bisexual flowers. (**A**–**C**) Branch of an inflorescence from the WT (**A**), L16 (**B**), and L6 (**C**) plants. For clarity, the sepals and petals were removed from the flowers. Yellow arrows indicate male flowers; white arrow indicates female flower; and red arrows indicate bisexual flowers; scale bars = 1 cm. (**D**) Cytokinin levels in male and female flowers of WT plants. The values represent the means ± standard deviations (*n* = 3). Student’s *t*-test was used for the statistical analyses: * *p* ≤ 0.05, ** *p* ≤ 0.01. (**E**) Female flowers of the WT plants and bisexual flowers of the L16 and L6 plants after the sepals and petals were removed. The inset shows the stamens marked by red ellipse; scale bar = 3 mm.

*J. curcas* is monoecious with both male and female flowers born from the same inflorescence [4]. Most of the flowers on the WT inflorescences were male, and female flowers only accounted for 7.63% of the total flowers (Figure 4A and Table 1). However, the *JcTM6:AtIPT4* transgenic plants showed altered sex expression. L16 produced male and bisexual flowers, which accounted for 42.81% and 57.19% of the total flowers, respectively (Figure 4B and Table 1). In the L6 line, the inflorescences presented higher cytokinin contents, and all the flowers were bisexual (Figure 4C and Table 1). The percentage of pistil-containing flowers was positively correlated with the cytokinin content, which indicated that a higher endogenous cytokinin content in inflorescences switched the sex expression balance from maleness to femaleness. Correspondingly, in WT *J. curcas*, the levels of bioactive cytokinins (iP and tZ) in female flowers were significantly higher than those in male flowers (Figure 4D). These results suggest that cytokinin promotes pistil development.

In the bisexual flowers of transgenic *J. curcas*, the pistil was located in the center, and its base is surrounded by stamens (Figure 4E). During *J. curcas* female flower development, both the pistils and the stamens emerge at the early stages, and the stamens abort during subsequent stages; however, no emergence of female tissues was detected during male flower development [44]. Together with no trace of female tissues emerging in the male flowers of the L16 transgenic line, we thus hypothesized that the bisexual flowers derived from the female flower primordia.

### 2.5. Flowers of JcTM6:AtIPT4 Transgenic J. curcas Developed Abnormally

**Figure 5:**
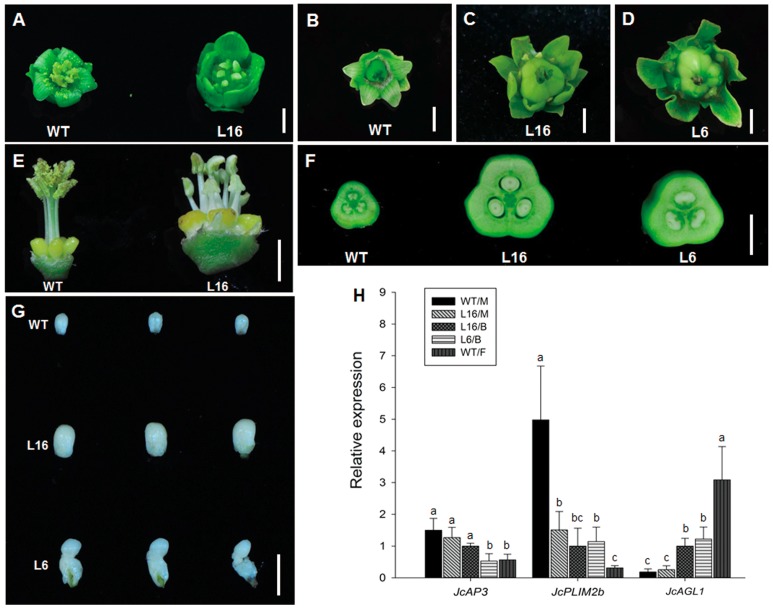
The flowers of *JcTM6:AtIPT4* transgenic *J. curcas* developed abnormally. (**A**) Male flowers of WT and L16 plants. (**B**) Female flower of WT plants. Bisexual flowers of L16 (**C**) and L6 plants (**D**). (**E**) Male flowers of WT and L16 plants with the sepals and petals removed. (**F**) Cross-sections of ovaries of WT, L16, and L6 plants. (**G**) Ovules of WT, L16, and L6 plants. All scale bars = 3 mm. (**H**) Expression of genes involved in floral development in the WT male flowers (WT/M) and female flowers (WT/F), L16 male flowers (L16/M), L16 bisexual flowers (L16/B), and L6 bisexual flowers (L6/B). The values represent the means ± standard deviations (*n* = 3). Student’s *t*-test was used for the statistical analyses. Different letters (a, b and c) indicate significant differences (*p* ≤ 0.05).

Because the *JcTM6* promoter directed the expression of the transgene in the male and female flowers in *J. curcas*, the transgenic and WT plants also exhibited differences in floral organ development. As shown in Figure 5A–D, the transgenic lines produced larger flowers than the WT plants. In the L16 line, the male flowers had an increased size (Figure 5A and Figure S1A), and the phenotype of the stamens was significantly different from that of the WT stamens. In general, the stamens of WT *J. curcas* are diadelphous and arranged in two tiers of five each (Figure 5E). In addition, the inner tier is united, and the outer tier is free [45]. However, the stamens in male flowers of the L16 line displayed an expanded arrangement without forming two bundles, similarly to distinct stamens (Figure 5E). Moreover, none of the stamens produced any visible pollen. The analysis of the bisexual flowers of the L16 and L6 lines revealed that the two outer whorls of sepals and petals were also larger than those of the WT female flowers with the exception that the L6 petals were smaller in size (Figure S1B). The stamens in the third whorl exhibited abnormal development, particularly in the L6 line. In addition, the growth of the pistil in the fourth whorl was markedly enhanced (Figure 4E and Figure 5C,D), which could result from the high expression of *AtIPT4* in pistils driven by the *JcTM6* promoter. An analysis of the cross-section of the ovaries revealed that the locules and ovules of the transgenic lines exhibited normal development, similarly to those of the WT plants (Figure 5F). However, separation of the ovules from the locules revealed that the ovules of the L6 line were deformed (Figure 5G), which indicated that high cytokinin levels impaired ovule development. Thus, both the stamens and pistils in the bisexual flowers were infertile. In addition, although the flowers in the transgenic lines exhibited abnormal development, the numbers of floral organs in each whorl were the same as those in the WT flowers.

Based on the severe phenotypic changes in the stamens and pistils, we hypothesized that the elevated cytokinin levels gave rise to alterations in the expression of development-related genes in the transgenic lines. We thus examined the expression of the *J. curcas APETALA3* (*JcAP3*) and *PLIM2b* (*JcPLIM2b*) genes, which are involved in stamen development, and the *J. curcas AGAMOUS-like 1* (*JcAGL1*) gene, which is involved in pistil development, in the flowers of the transgenic and WT plants, respectively. As shown in Figure 5H, the expression levels of *JcAP3* in the male and bisexual flowers of the L16 line were almost the same as those in the male flowers of WT plants. However, in the bisexual flowers of the L6 line, *JcAP3* expression was reduced to the levels found in the female flowers of the WT plants. This finding is consistent with the severely abnormal phenotype of the L6 stamens. However, because *JcPLIM2b* is involved in pollen development [46], its expression was significantly downregulated in both the L16 and L6 lines, which implies pollen defects in the transgenic lines. In accordance with the abnormal phenotype of the pistils in the bisexual flowers, the expression levels of *JcAGL1* were also significantly decreased in the transgenic lines compared with the levels found in the WT female flowers. These results suggested that an overdose of cytokinins could repress the expression of some floral development genes in *J. curcas*.

## 3. Discussion

Several studies have suggested that exogenous cytokinins treatment affects flower development in monoecious *J. curcas* [40,41,47,48]. To investigate the effect of endogenous cytokinins on *J. curcas* flower development, the native *J. curcas JcTM6* promoter, which shows predominant activity in flowers, was used to drive the expression of the cytokinin biosynthetic gene *AtIPT4* in this study. As expected, the number of flowers was significantly increased in the *JcTM6:AtIPT4* transgenic plants, which is consistent with the findings obtained after the exogenous application of cytokinins. Because cytokinins promote cell division in the shoot apical meristem, we hypothesize that the elevated cytokinin levels in the inflorescences increased the size of the inflorescence meristems, resulting in the formation of more floral primordia. Evidence showing that increased cell division in cytokinin-overproducing Arabidopsis contributes to a large inflorescence with more floral primordia confirmed that cytokinins enhance proliferation of the inflorescence meristem [18,19].

Cytokinin also plays an important role during floral organ development, particularly pistil development. The *Shy Girl* (*SyGI*) gene, which encodes a C-type ARR, a negative regulator of cytokinin signaling [7,49], has been identified as a male sex-determining gene in dioecious *Actinidia*. The expression of *SyGI* in Arabidopsis and *Nicotiana* suppresses pistil development without affecting male development [50]. In this study, we assessed cytokinin signaling during floral development in Arabidopsis using the *TCSn:GFP* marker [51], and found that strong signals were detected in the central zones of floral primordia (Figure S2A–C) and during pistil development (Figure S2D–F). Similarly, as shown in *J. curcas*, pistil growth requires more bioactive cytokinins (Figure 4D). Consequently, the higher cytokinin levels in *JcTM6:AtIPT4* transgenic *J. curcas* induced the production of pistils in most and even all of the flowers, whereas fewer flowers in the WT plants produced pistils. All the flowers with pistils in the transgenic plants were bisexual rather than female. This finding differs from the observed increases in both female and bisexual flowers after the exogenous application of cytokinins, and bisexual flowers accounts for a low proportion of the flowers [40,41]. However, the number of bisexual flowers is positively correlated with the development of inflorescence to be treated by cytokinin [41]. In transgenic *J. curcas*, the *JcTM6* promoter drives the expression of *AtIPT4* from inflorescence emergence to floret anthesis, which showed a phenotype of bisexual flowers induction instead of more female flowers. The previous study showed that *J. curcas* female flowers are unisexual with aborted stamens [44]. Our results implied that the degenerated stamens could be rescued by excess cytokinins during female flower development.

The bisexual flowers in transgenic *J. curcas* were more feminine due to the presence of a huge ovary surrounded with puny stamens (Figure 4E). Because the *JcTM6* promoter was capable of driving the expression of *AtIPT4* at a high level during pistil development (Figure 1B), the pistil-localized overproduction of cytokinins was hypothesized to induce ovary enlargement. In Arabidopsis, the overproduction of cytokinins or the enhancement of cytokinin signaling in flowers also results in oversized floral organs with increased cell numbers [18,19,52]. Unexpectedly, all the bisexual flowers as well as the male flowers in the transgenic plants were infertile. During female reproductive development in *J. curcas*, the cytokinins (zeatin) content at the early developmental stage is significantly increased compared with that in inflorescence primordia, whereas a substantially reduced level is observed at the later developmental stages [31]. This finding suggests that a higher cytokinin content is required for early pistil development, whereas a lower cytokinin content is required for late pistil development. Therefore, the consistently high levels of cytokinins during female development could give rise to infertility. In accordance with this finding, the pistil development-related gene *JcAGL1* was downregulated in the bisexual flowers (Figure 5H).

Although a visual analysis indicated that the stamens grew from the base of the ovary, the stamens, particularly those in the L6 line, developed abnormally (Figure 4E). The analysis of the transgenic line L16 showed that the morphology of the stamens in male and bisexual flowers was similar, and both of these flowers were sterile due to pollen abortion. In general, cytokinins are important for male reproductive development. Stamen fertility is impaired by an excessive or a too-low cytokinin content [53,54,55]. In *J. curcas*, the pattern of cytokinin production during male reproductive development is opposite to that observed during female reproductive development [31], which indicates that low and high levels of cytokinins are beneficial for early and late stamen development, respectively.

In addition, altered floral organs are caused by increased cytokinin levels in Arabidopsis [18,19], but the number and position of floral organs in the flowers of the transgenic *J. curcas* plants were as well defined as those in the WT plants. This result suggested that the ectopic overproduction of cytokinins in the transgenic *J. curcas* flowers did not impair the expression of genes involved in the determinacy of the floral meristem. *AG* has been identified as a key regulator of floral meristem determinacy, and the maintenance of its expression at the center of the meristem mediates the expression of other genes that promote floral meristem determinacy [56,57,58]. The Arabidopsis *ag-1* mutant exhibits a flower-in-flower phenotype because the flowers constantly produce new sepals and petals [59]. Thus, the expression of *JcAG* in the transgenic lines was also examined. In agreement with our hypothesis, the *JcAG* expression level in the transgenic flowers was not markedly different from that in the WT flowers (data not shown), which implied that the floral meristem determinacy was not affected.

In conclusion, our study indicates that an increase in the endogenous cytokinin levels in *J. curcas* flowers is capable of enhancing inflorescence meristem activity and triggering female sex expression, which indicates that high-yielding *J. curcas* could be bred by modifying the cytokinin levels in flowers. This is the first time to modify the flower number and sex expression by regulating the endogenous cytokinins in *J. curcas*. Because continuously high cytokinin levels during female flower development could give rise to infertility, fine-tuning both the cytokinin contents and the spatiotemporal distribution of cytokinins is necessary. For example, *JcAP1* promoter, which was predominantly active at the early stages of flower development [60], would be a better choice to induce the expression of cytokinin biosynthetic genes in transgenic *J. curcas* in further study.

## 4. Materials and Methods

### 4.1. Plant Materials

The *J. curcas* plants used in this study were cultivated in Xishuangbanna, Yunnan Province, China, as described previously [40]. TCSn:GFP transgenic Arabidopsis, which was kindly provided by Bruno Müller (University of Zurich, Zurich, Switzerland), was grown at 22 °C with a 16-h light/8-h dark photoperiod.

### 4.2. Plasmid Construction

A 1.8-kb 5′ flanking region of *JcTM6* was amplified from *J. curcas* genomic DNA using the following primers: Forward (5’-tctagaAATAGCTATAAAATCAATT-3’) and reverse (5′-ggatccTTTTCCTTTCTTCTTGATA-3′). To generate the *JcTM6:GUS* fusion, pBI101 [61] and the pGEM-T Easy vector containing the 1.8-kb *JcTM6* promoter were digested with *Xba*I (lowercase letters in the forward primer) and *Bam*HI (lowercase letters in the reverse primer). The two fragments were linked using T4 ligase (Shanghai Promega, Shanghai, China). *AtIPT4* (GenBank accession no. AT4G24650) was amplified from the *AP1:AtIPT4* construct [18] using the following primers: Forward (5′-cccgggCTCAATTTACGACATGAAGTG-3′) and reverse (5′-gagctcGTTTTGCGGTGATATTA GTC-3′). To generate the *JcTM6:AtIPT4* construct, the *JcTM6:GUS* plasmid and the pGEM-T Easy vector containing AtIPT4 were digested with *Sma*I (lowercase letters in the forward primer) and *Sac*I (lowercase letters in the reverse primer), and the two fragments of the *JcTM6* promoter and *AtIPT4* were linked using T4 ligase (Shanghai Promega, Shanghai, China). The resulting constructs of *JcTM6:GUS* and *JcTM6:AtIPT4* were then transferred to *Agrobacterium tumefaciens* EHA105 for *J. curcas* transformation.

### 4.3. Plant Transformation

The method used for *J. curcas* transformation was described previously [62].

### 4.4. Histochemical and Fluorometric GUS Assay

For histochemical GUS staining, various plant organs of transgenic *J. curcas* were incubated overnight at 37 °C in GUS assay buffer with 50 mM sodium phosphate (pH 7.0), 0.5 mM K_3_Fe_6_, 0.5 mM K_4_Fe_6_·3H_2_O, 0.5% Triton X-100, and 1 mM X-Gluc and then cleared in 70% ethanol [61]. The samples were examined via stereomicroscopy (Leica M80). To examine the activity of the *JcTM6* promoter in different plant organs, a fluorometric GUS assay according to the protocol described by Jefferson, Kavanagh, and Bevan [61] with the modification that 2 mM MUG was added to the reaction buffer. The fluorescence was determined using a Gemini XPS microplate spectrofluorometer (Molecular Devices Corporation, Sunnyvale, CA, USA). The protein concentrations of the plant extracts were measured using the Bradford method [63].

### 4.5. Quantification of CKs

Two-week-old inflorescence buds from wild-type and transgenic *J. curcas* and male and female flower buds from WT *J. curcas* were collected and immediately placed in liquid nitrogen. The cytokinins were extracted and quantified using the polymer monolith microextraction/hydrophilic interaction chromatography/electrospray ionization tandem mass spectrometry method as described previously [64]. Three independent biological replicates and three technical replicates were measured for each sample.

### 4.6. qRT-PCR Analysis

The expression levels of genes in the inflorescences and flowers of the WT and transgenic *J. curcas* plants were examined by quantitative reverse transcriptase-polymerase chain reaction (qRT-PCR). Total RNA was isolated [65] and reverse transcribed using the PrimeScript^®^ RT reagent kit with gDNA Eraser (Takara , Dalian, China). qRT-PCR was performed using LightCycler^®^ 480 SYBR Green I Master Mix with the Roche 480 real-time PCR detection system (Roche Diagnostics, Indianapolis, IN, USA). All gene expression data obtained in the qRT-PCR assay were normalized to the expression of *JcActin1* [66]. The primers used in the qRT-PCR assay are listed in Table S1.

### 4.7. Tissue Preparation for Confocal Analysis

For tissue preparation, inflorescences were collected, immediately placed in 2.5% paraformaldehyde (PFA) (BBI) (pH 7.0) on ice, vacuum infiltrated for 30 min, and stored overnight at 4 °C. The fixed samples were washed with 10% sucrose and 1% PFA (pH 7.0) for 20 min, with 20% sucrose and 1% PFA (pH 7.0) for 20 min, and with 30% sucrose and 1% PFA (pH 7.0) for 30 min. The samples were then embedded in 5% to 7% LM agarose (Shanghai Promega, Shanghai, China) liquid gel at 30 °C and solidified by incubation at 4 °C for 15 min. Sections with a thickness of 40 to 70 μm were prepared using a Leica VT1000S vibratome. To obtain high-resolution images, the samples were stained with 50 μg/mL PI (Sigma-Aldrich, Shanghai, China) [67].

### 4.8. Confocal Microscopy

Images were obtained using an Olympus FV1000 confocal microscope. The GFP signal was detected using excitation and emission wavelengths of 488 and 500–530 nm, respectively. For the detection of PI staining, excitation was performed using a 543 nm laser line, and emission was determined at 550–618 nm.

## Figures and Tables

**Table 1:** Numbers of male, female, and bisexual flowers per inflorescence in the WT plants and the transgenic lines L16 and L6.

	Total Flowers	Male Flowers	Female Flowers	Bisexual Flowers
WT	180.25 ± 39.20	166.50 ± 35.94 (92.37%)	13.75 ± 4.68 (7.63%)	0.00
L16	344.00 ± 58.42	147.25 ± 37.69 (42.81%)	0.00	196.75 ± 55.06 (57.19%)
L6	767.00 ± 163.31	0.00	0.00	767.00 ± 163.31

The values represent the means ± standard deviations (*n* = 8). The percentages in the brackets indicate the corresponding percentages of male, female and bisexual flowers among the total flowers.

## Supplementary Materials

Supplementary materials can be found at https://www.mdpi.com/1422-0067/21/2/640/s1.

## Abbreviations

AG	AGAMOUS
AGL1	AGAMOUS-like 1
AHK	ARABIDOPSIS HISTIDINE KINASE
AP1	APETALA1
AP3	APETALA3
ARR	ARABIDOPSIS RESPONSE REGULATOR
6-BA	6-benzylaminopurine
CKX	cytokinin oxidases/dehydrogenase
cZ	cis-zeatin
cZR	cis-zeatin riboside
DAP	days after pollination
GUS	β-glucuronidase
iP	isopentenyladenine
iPR	isopentenyladenosine
IPT	isopentenyltransferase
LOG	LONELY GUY
STM	SHOOT MERISTEMLESS
SyGI	Shy Girl
TDZ	thidiazuron
TM6	TOMATO MADS BOX GENE 6
tZ	trans-zeatin
tZR	trans-zeatin riboside
