# Supplementary material for: Flower-Specific Overproduction of Cytokinins Altered Flower Development and Sex Expression in the Perennial Woody Plant Jatropha curcas L
Source: Int J Mol Sci. 2020 Jan 18;21(2):640. doi: 10.3390/ijms21020640 (PMC7013397; doi:10.3390/ijms21020640)
Supplement: Supplementary file 1 [file ijms-21-00640-s001.pdf]

International Journal of
Molecular Sciences

Supplementary Material

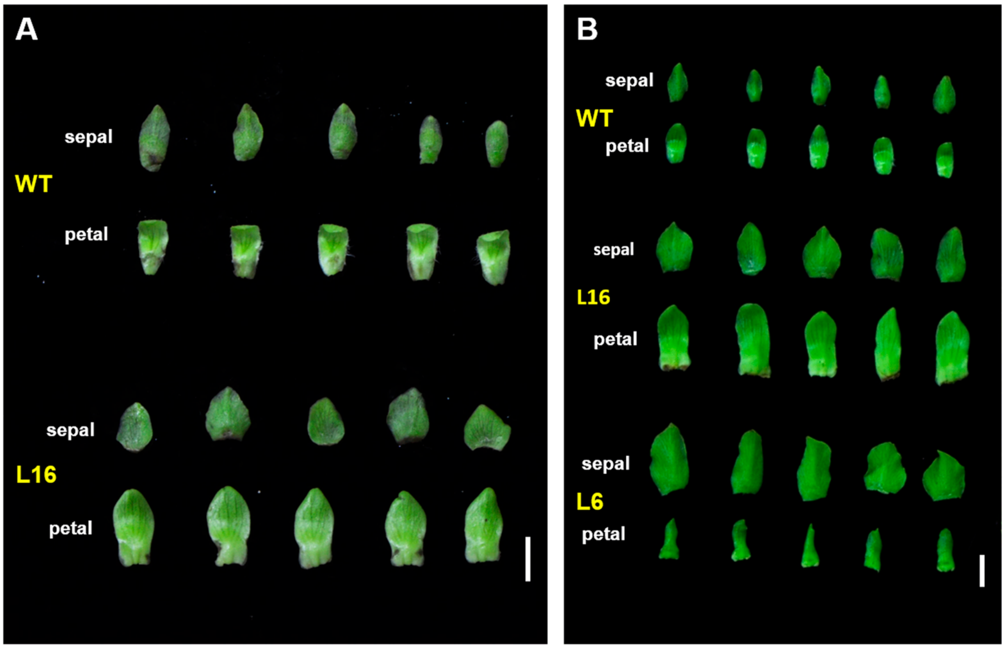

Figure S1. The sepals and petals of transgenic *Jatropha* developed abnormally. (A) Sepals and petals from the male flowers of WT and L16. (B) Sepals and petals from the female flowers of WT and the bisexual flowers of L16 and L6; scale bars = 5 mm.

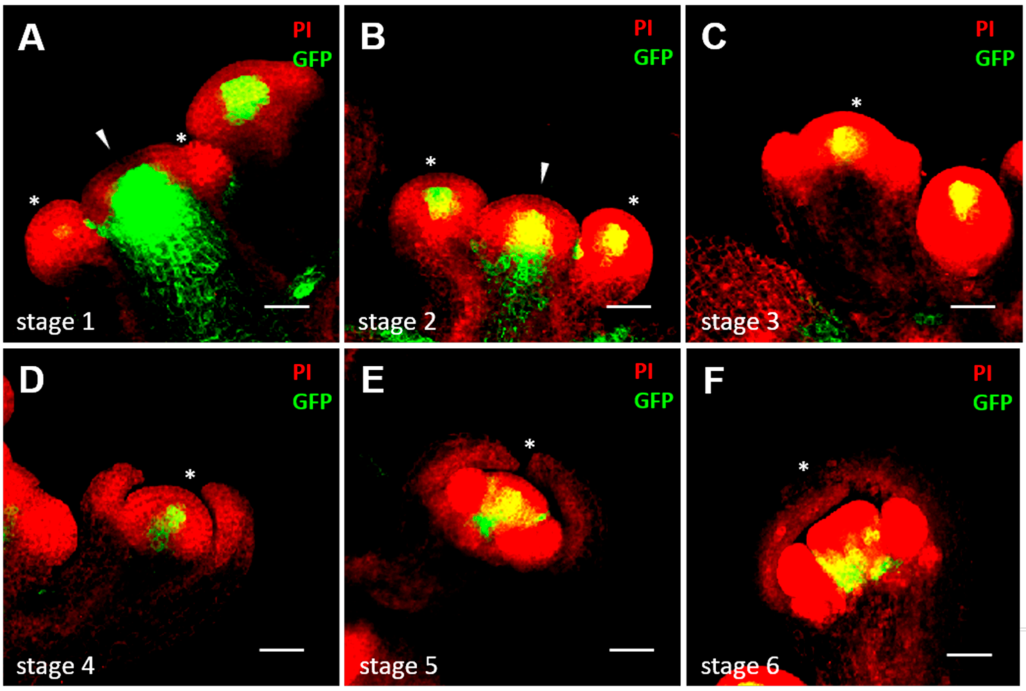

Figure S2. The central regions of the floral primordium in Arabidopsis showed strong cytokinin signalling. (A–F) Longitudinal section of floral primordia of the *TCSn:GFP* transgenic Arabidopsis stained by propidium iodide (PI) (red) from stage 1 (A) to stage 6 (F). Asterisk, floral primordium; arrowhead, inflorescence meristem; scale bars = 30μm.

Table S1. List of primers used in the qRT-PCR analysis.

Gene	Forward	Reverse
AtIPT4	5′-TCGCTGAGTTCCACCGCTCTAAG-3′	5′-AGGGTCCCATTTATCCATGTCATTG-3′
JcActin	5′-CTCCTCTCAACCCCAAAGCCAA-3′	5′-CACCAGAATCCAGCACGATACCA-3′
JcAHK2	5′-TAGCGAGAAACTGGGATTTAGCA-3′	5′-TTCGTGATTGGCTTATTAGTGGG-3′
JcARR3	5′-CAGTTGCTGCTCATTTCATTCGTC-3′	5′-ATCTCCTTCGCCGTCGCCTTTA-3′
JcAP3	5′-AGCCCTACAACATCGACAAA-3′	5′-TCCAGACTCCTCATCTCGTC-3′
JcPLIM2b	5′-AGGGAGCTATACCCATGTCC-3′	5′-CATCTGCCAACCCAACAAAAA-3′
JcAGL1	5′-TTTTGCAAGCGCAGAAATGG-3′	5′-CCTGATTTGCCTGCGTAGTT-3′
